# Recovery following Orthognathic Surgery Procedures—A Pilot Study

**DOI:** 10.3390/ijerph192316028

**Published:** 2022-11-30

**Authors:** Cristian Dinu, Avram Manea, Denisa Tomoiagă, Mihaela Băciuț, Oana Almășan, Andrei Otto Mitre, Ioan Barbur, Mihaela Hedeșiu, Gabriel Armencea, Horia Opriș, Sebastian Stoia, Tiberiu Tamaș, Grigore Băciuț, Florin Onișor, Simion Bran

**Affiliations:** 1 Department of Maxillofacial Surgery and Implantology, University of Medicine and Pharmacy “Iuliu Hațieganu”, 37 Cardinal Iuliu Hossu Street, 400029 Cluj-Napoca, Romania; 2 Department of Prosthetic Dentistry and Dental Materials, Iuliu Hațieganu University of Medicine and Pharmacy, 32 Clinicilor Street, 400006 Cluj-Napoca, Romania

**Keywords:** orthognathic surgery, edema, trismus, pain, anesthesia, hypoesthesia, recovery after surgery

## Abstract

This study aims at evaluating and categorizing patients’ objective and subjective postoperative recovery symptoms after bimaxillary orthognathic surgery assigning the healing process. The patients were monitored throughout the recovery process, and their symptoms were managed. A prospective, observational study was performed. Patients with Class II and III malocclusion (aged 18 to 35) were evaluated and monitored preoperatively, and postoperatively at 48 h, 2 weeks, 1 month, and 3 months postsurgery. A questionnaire was used to assess pain and anesthesia/hypoesthesia. The most common objective and subjective signs that were correlated with the healing process were edema, hematoma, trismus, pain, and anesthesia/hypoesthesia. Edema peaked at 48–72 h postoperatively (distance between eye’s external canthus and gonion, mean difference = 4.53, between tragus and cheilion, mean difference = 7, between tragus and gnathion, mean difference = 4.65, *p* < 0.001); mouth opening amplitude was significantly decreased during the first two weeks postsurgery (class II, mean difference = 32.42, *p* = 0.006, class III, mean difference = 44.57, *p* < 0.001), but it steadily and considerably improved over three months. The nose tended to widen postsurgery. The most severe pain experienced by patients was of medium intensity in the mandibular body, described as pressure, and usually did not spread. Patients were most severely and persistently impacted by anesthesia/hypoesthesia.

## 1. Introduction

Anomalies of the craniomaxillofacial complex are the expression of changes occurring in the growth and development of various segments of the neurocranium or viscerocranium during the intrauterine period and throughout the entire somatic development. They can be visible at birth due to the suffering of the embryo during intrauterine life, but they can also appear during life through the action of different etiological factors, whereas dental anomalies are defined by any form of interruption of the eruption process of a tooth germ from its initial position of development in the alveolar bone into its functional position, of the oral cavity [1]. Individuals with extensive malocclusion have much worse mastication function, aesthetics, general oral health, periodontal disease, and general self-esteem, all of which can be treated through orthognathic surgery and consecutive specific treatments [2]. By properly planning and carrying out precise orthodontic planning, intraoperative surgical procedures, and postoperative procedures, long-term three-dimensionally stable occlusion results can be attained [3].

The correct repositioning of the maxilla, mandible, or even both can be accomplished through orthognathic surgery [4]. The most common surgical procedure in the maxilla is the LeFort I osteotomy (a low horizontal maxillary osteotomy). It allows for 3D movements of the maxilla, including translational and rotational displacements, and even transversal deficit correction when multisegmental osteotomies are employed [5,6]. Often, it is indicated in conjunction with the bilateral sagittal split osteotomy (BSSO), the most used orthognathic technique in the mandible for correcting Class II or III malocclusion and other discrepancies. The BSSO osteotomy splits the ramus and the posterior body of the mandible sagittaly, which allows for the complex movement of the mandible [7].

Orthognathic surgery may be the best option for treating significant hyperdivergent Class II skeletal malocclusions in mature individuals [8]. The lower-jaw strategy has replaced the maxilla-first technique in some situations as the preferred method of therapy for bimaxillary orthognathic surgery due to the development of robust screw stabilization and the need to avoid any potential inaccuracies that can occur throughout the initial occlusal recording [9]. After Class II and III surgical orthognathic procedures, considerable intended vertically mandible alterations are accomplished. Some degree of relapse was observed from a vertical point of view with these patients over the long term [10].

Congenital and acquired dentofacial abnormalities are routinely treated with orthognathic surgery; hence, the appearance of every facial feature, such as the nose, is impacted through any surgical intervention performed to alter or restore facial characteristics [11]. Since orthognathic surgery procedures are accompanied by swelling, pain, and peripheral nerve abnormalities following orthognathic surgery, low-level laser therapy was helpful in reducing discomfort, edema, and neurosensory disturbances involving the inferior alveolar nerve [12]. Although it had no effect on peripheral nerve abnormalities, supplying corticosteroids after orthognathic surgery enhanced the reduction in face edema [13]. Hilotherapy, which involves using a face mask to apply cool pressure at a controlled temperature, was linked with considerable decreases in postoperative facial pain and edema [14]. In lowering postsurgical edema after mandible orthognathic surgery, photobiomodulation treatment was the most beneficial addition to oral nonsteroidal anti-inflammatory drugs [15].

This study aims at evaluating and classifying the patient’s objective and subjective postoperative recovery symptoms after bimaxillary orthognathic surgery into stabilizing parameters and assigning the healing process, monitoring parameters over time during checkup visits for persistence, amplification, or symptom relief, observing changes throughout the course of the recovery process, classifying relevant data into predicable categories that enable patients’ symptoms to be better understood in terms of their nature, severity, and duration, determining if there is a correlation between the severity of remission symptoms and signs evolving over time in patients who presented with a Class II or III malocclusion, and observing changes in the recovery process. This information is extremely valuable to practitioners because it allows for them to not only provide more accurate data to their patients, but also to develop strategies aimed at reducing their patients’ discomfort.

## 2. Materials and Methods

A prospective, observational study was performed between July and September 2021. The study was approved by the ethics committee of the University of Medicine and Pharmacy in Cluj-Napoca (approval number 248/30.06.2021). A three-month study was conducted. Each patient was informed on the study protocol, the procedures for collecting the necessary data for the study, the time frame for completion, and the number of assessments the evaluator was required to complete. By filling out and signing the legal paperwork addressing the acquisition, processing, storage, and privacy protection of personal data, patients gave their informed consent to participate in the study. For each patient, a file was created with the qualitative data provided by the patient and recorded in their evaluation form before surgery at 48 h, 2 weeks, and 1 and 3 months after surgery combined with the objective information determined by the evaluator at the following time intervals: preoperatively, and postoperatively at 48 h, 2 weeks, 1 month, and 3 months.

Patients needing bimaxillary orthognathic surgery (LeFort I and BSSO—Epker Technique) were included in the present study. Patients with malocclusions that did not involve orthognathic surgery and those who had only undergone monomaxillary or segmental surgery were excluded.

Five parameters were evaluated: three objective and two subjective. The three main objective parameters that were evaluated were edema, mouth opening amplitude, and nose width, whereas pain and anesthesia/hypoesthesia were the two subjective evaluated parameters. A digital caliper and ruler were used as the measuring tools to ascertain and quantify the objective data, and all the results were recorded in millimeters. Subjective data were collected using a nonvalidated operator-assisted survey that included questions on all relevant parameters of interest.

**Figure 1:**
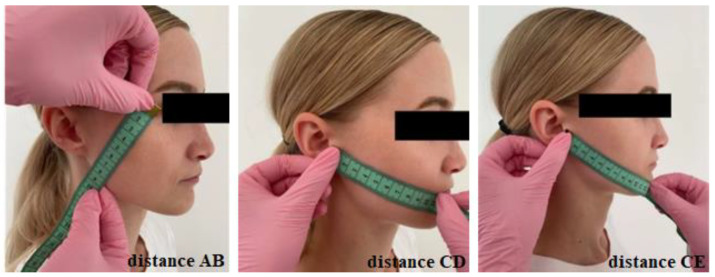
Edema measurement. Distance AB (between the eye’s external canthus and gonion), distance CD (between tragus and cheilion), and distance CE (between tragus and gnathion).

The objective parameter of edema was defined as the distance between the external canthus, gonion, tragus, cheilion, and gnathion of the eye, and was measured with a flexible ruler. Three distances were established between these points: distance AB (between the eye’s external canthus and gonion), distance CD (between tragus and cheilion), and distance CE ((between tragus and gnathion) (Figure 1).

**Figure 2:**
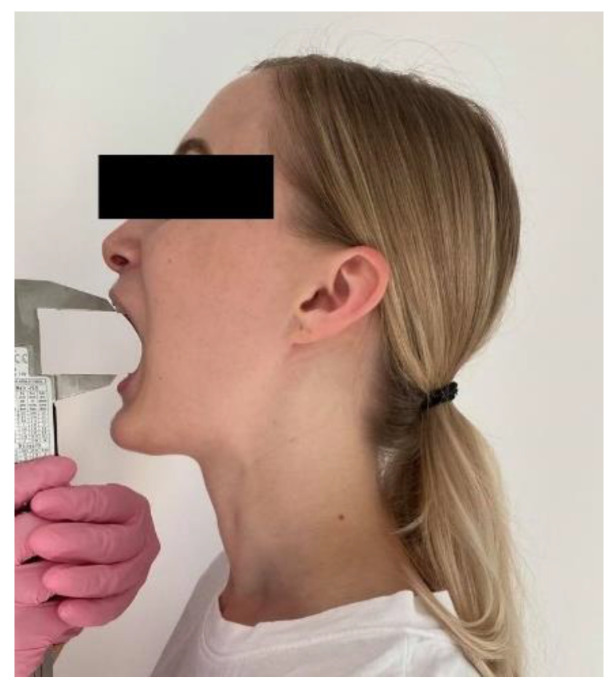
Mouth opening measurement.

The mouth’s opening was measured with a digital caliper, and the quantitative value (length) of the mandibular path was calculated. At the maximal opening, the distance between the right central upper incisor’s incisal edge and a tangent to the lower right central incisor’s incisal edge was measured (Figure 2).

**Figure 3:**
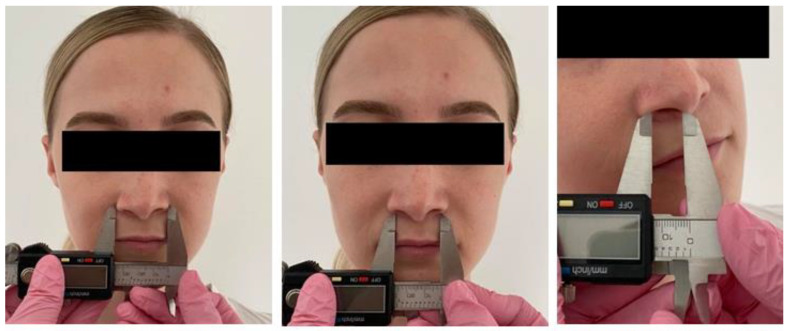
Nose width measurement.

The nose’s width indicates soft-tissue changes after the surgery. It was examined with the use of the digital caliper. Anthropometric points alare, the most external point of the nose’s wings, the distance between the insertions of the nasal fins, and the diameter of the left and right nostrils were all measured (Figure 3).

A self-developed, nonvalidated questionnaire based on previous research was used to assess pain [16,17]. The survey inquired about the affected region, the degree of pain as judged by the patient using a numerical rating scale ranging from 0 to 10 (where 0 represented no pain and 10 represented pains felt to the greatest extent possible), the type of pain (which was classified as pressure, pulsating, stabbing, flashing, tingling, or twitching), and whether pain extended to other structures.

Anesthesia/hypoesthesia was investigated because the elongation of the inferior alveolar nerve branches occurs during the BSSO via bone segment manipulation. It was graded on a scale from 1 to 5, where 1 represented an unmodified tactile experience, 2 slightly modified, 3 moderate sensation, 4 almost absent, and 5 was the absence of tactile sensation. Statistical analysis was performed using GraphPad Prism software, version 8. The Shapiro–Wilk test was used to determine the normality of the data distribution for continuous quantitative data; a *p*-value of <0.05 suggested a normal distribution. The variables were investigated according to how they fluctuated over time (both before surgery and at different postoperative intervals); consequently, they were considered to be dependent variables. The ANOVA test was employed for the analysis of more than two groups in accordance with a single independent variable (the time variable). The Bonferroni post hoc test was used for comparison between pairs of groups. A two-way ANOVA test was used for the study of more than two groups with two independent variables (time variable and skeletal class). The Tukey test was used to compare the group means when there was a statistically significant association between the time variable and the continuous dependent variable. The between-group analysis of ordinal and nominal quantitative variables was conducted using the Friedman test. When comparing groups, Dunn’s mean rank test was used if the test had statistical significance. The tests were considered to be statistically significant with an error threshold of 5% (*p*-value < 0.05).

## 3. Results

A total of 13 patients were examined (five males and eight females). Patients ranged in age from 18 to 35 years old, were of both sexes, and had dentomaxillary Class II and III malocclusion types (Table 1).

**Figure 4:**
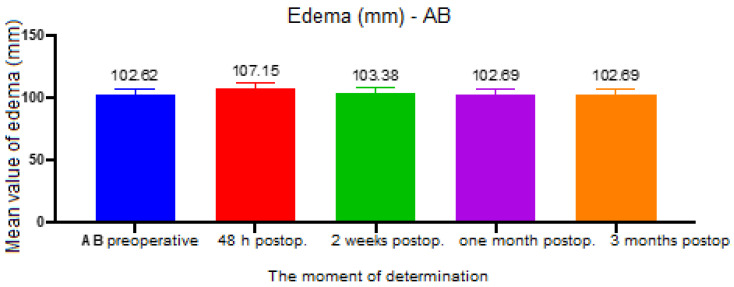
Edema assessment, AB distance.

All continuous variables considered for the objective parameter edema were considered to be normally distributed (Shapiro–Wilk *p* > 0.05), and there were statistically significant differences between time intervals for all dimensions measured. The ANOVA test yielded a *p* < 0.001 value between the AB points (eye’s external canthus and gonion), and the assessment among groups revealed a statistically significant difference between the preoperative and 48 h postoperative groups (*p* < 0.001, mean difference = 4.53), 48 h postoperative and 2 weeks postoperative (*p* < 0.001, mean difference = 3.77), 48 h and 1 month postoperative (*p* < 0.001, mean difference = 4.46), and between 48 h and 3 months postoperative (*p* < 0.001, mean difference = 4.46) (Figure 4).

**Figure 5:**
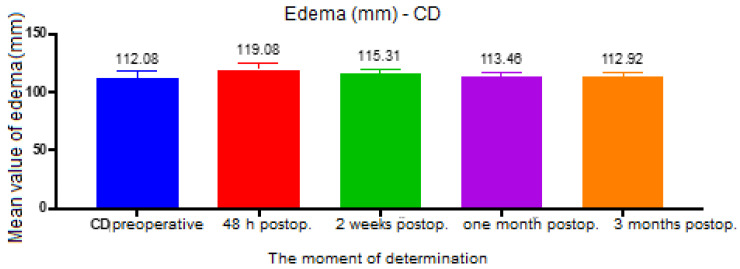
Edema assessment, CD distance.

For the CD distance (between tragus and cheilion), the ANOVA test had a value of *p* = 0.008, and the analysis between groups revealed a statistically significant difference between the preoperative and 48 h postoperative groups (*p* < 0.001, mean difference = 7), 48 h postoperative and 2 weeks postoperative (*p* = 0.002, mean difference = 3.76), 48 h and 1 month postoperative (*p* = 0.006, mean difference = 5.61) and between 48 h and 3 months postoperative (*p* = 0.006, mean difference = 6.15) (Figure 5).

**Figure 6:**
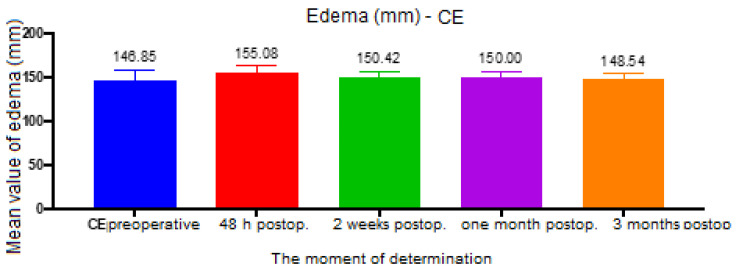
Edema assessment, CE distance.

For the CE distance (between tragus and gnathion), the ANOVA test had a value of *p* = 0.003, and the analysis between groups revealed a statistically significant difference between the preoperative and 48 h postoperative groups (*p* < 0.001, mean difference = 8.23), 48 h and 2 weeks postoperative (*p* < 0.001, mean difference = 4.65), 48 h postoperative and 1 month postoperative (*p* < 0.001, mean difference = 5.07), between 48 h and 3 months postoperative (*p* < 0.001, difference means = 6.53), and between 2 weeks and 3 months postoperative (*p* = 0.02, mean difference = 1.88) (Figure 6).

**Figure 7:**
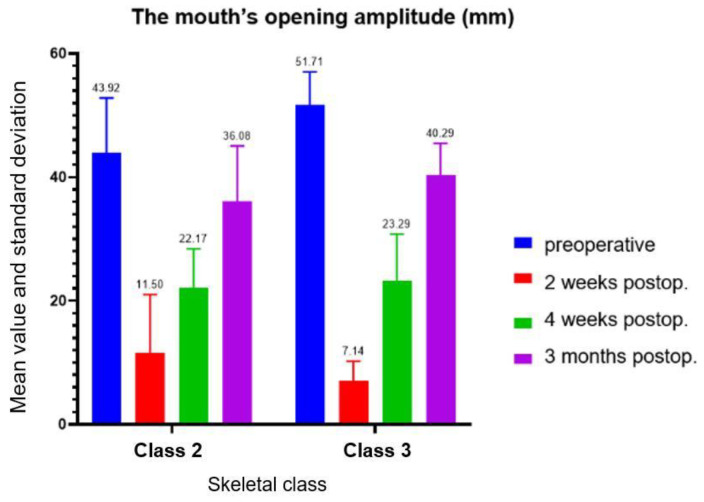
Mouth’s opening amplitude.

A two-way ANOVA test was used to analyze the mouth opening amplitude data that included, in addition to the moment of determination, the skeletal class (II or III). Taking the time variable into account, skeletal class had no effect on the amplitude of the mouth opening (*p* = 0.44). The time of determination had a significant impact on the results, with a statistically significant difference between the groups (*p* < 0.001). The test between groups was performed for each skeletal class individually. For Class II, there were statistically significant differences between preoperative and 2 weeks postoperative (*p* = 0.006, mean difference = 32.42), preoperative and 4 weeks postoperative (*p* = 0.02, mean difference = 21.75), 2 weeks postoperative and 3 months postoperative (*p* = 0.01, mean difference = 24.58) and between 4 weeks postoperative and 3 months postoperative (*p* = 0.02, mean difference = 13.92). For Class III, there were also statistically significant differences between all groups. Between preoperative and: 2 weeks postoperative (*p* < 0.001, mean difference = 44.57), 4 weeks postoperative (*p* < 0.001, mean difference = 28.43) and 3 months postoperative (*p* = 0.006, mean difference = 11.43). Between 2 weeks and 4 weeks postoperative (*p* < 0.001, mean difference = 16.14) and 3 months postoperative (*p* < 0.001, mean difference = 33.14) and between 4 weeks and 3 months postoperative (*p* < 0.001, mean difference = 17) (Figure 7).

**Figure 8:**
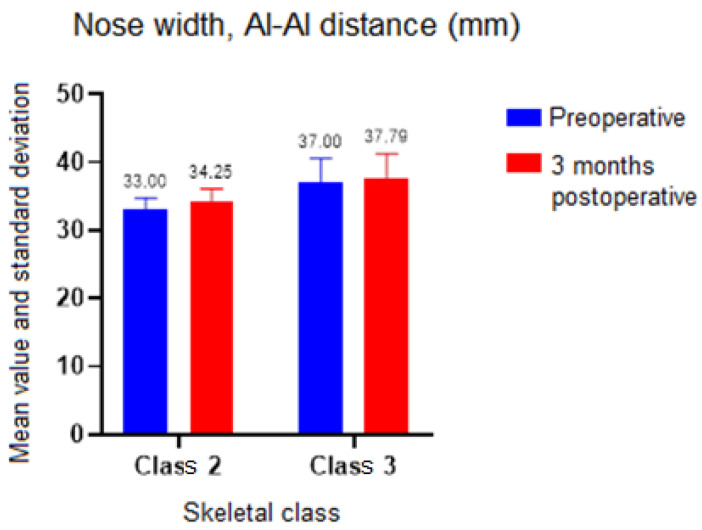
Nose width assessment.

In terms of nose width, for the Alare–Alare distance, both the skeletal class (*p* = 0.03) and the time of determination had an influence. When comparing classes, no statistically significant difference was detected between preoperative and 3 months postoperative (*p* = 0.08 for skeletal class II and *p* = 0.28 for skeletal class III). However, when the skeletal class was not considered, there was a difference between preoperative and 3 months postoperative (*t*-test *p* = 0.02, difference in means = 1). There was also a statistically significant difference (*p* = 0.04) of 4 mm in the preoperative moment between Skeletal Classes II and III (Figure 8).

**Figure 9:**
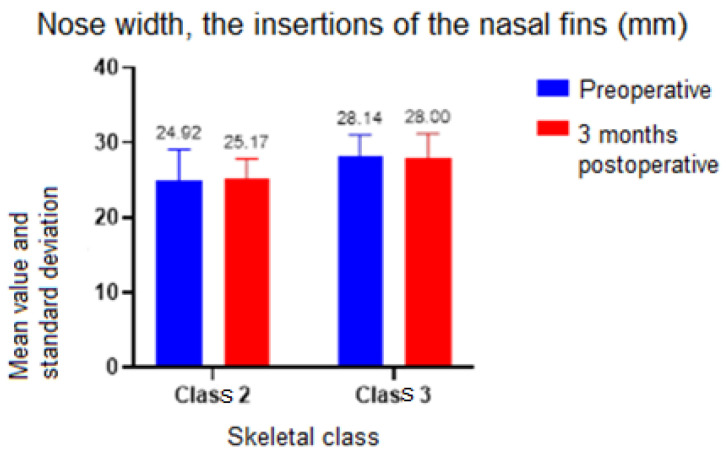
Nose width assessment, insertions of the nasal fins.

The time of determination (*p* = 0.91) and skeletal class (*p* = 0.11) had no statistically significant influence on the values for the distance between the insertions of the nasal fins. As a result, the group tests were no longer used (Figure 9).

**Figure 10:**
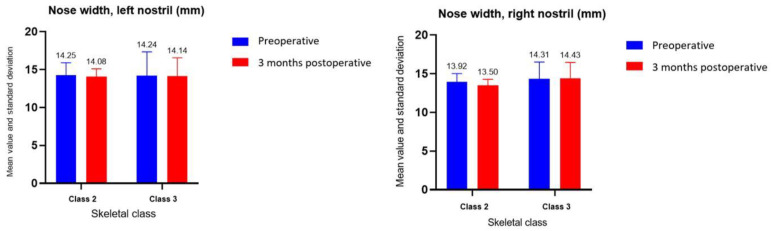
Nose width assessment, right and left nostrils.

In the case of the width of the nose, for the left nostril, the moment of determination (*p* = 0.58) and the skeletal class (*p* = 0.98) and for the right nostril, class (*p* = 0.49) and time of determination (*p* = 0.48) did not have a statistically significant influence on the values (Figure 10). Therefore, the group tests were not used.

**Figure 11:**
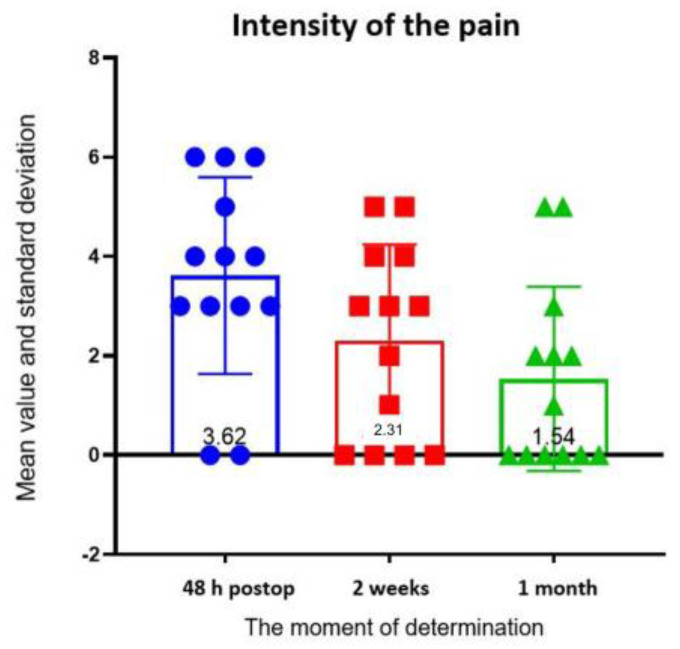
Pain intensity assessment.

The information gathered for pain evaluation revealed the following data about the affected area: at 48 h, the following areas were the most affected, in descending order: pain in the temporomandibular joint—1 patient, toothache—1 patient, and pain of the mandibular angle—1 patient. One month after surgery, seven patients reported no pain, three patients reported pain in the temporomandibular joint, two patients pain in the chin, and one patient pain in the cheeks. On a scale of 1 to 10, the patients rated the intensity of their pain as follows: 4 patients gave it a score of, 3 patients gave it a 6, 3 patients gave it a 4, one patient gave it a 5, and 2 did not present pain. One month after surgery, the intensity score for 3 patients was 2, 5 for 2 patients, 3 for 1 patient, and 0 for the remaining 6 patients. The Friedman test revealed that the distribution of pain intensity differed significantly between groups (*p* = 0.02). Dunn’s test revealed a statistically significant difference in pain intensity between 48 h and 1 month (*p* = 0.049) (Figure 11).

**Figure 12:**
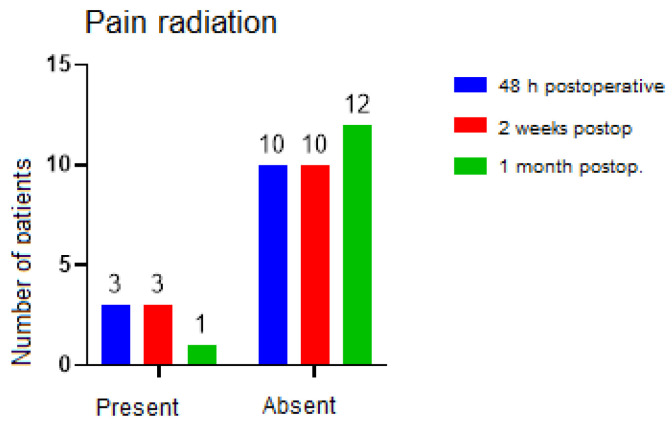
Pain radiation.

The codes observed for the type of pain at 48 h were pressure for 9 of the patients, pulsating pain for 2 patients, throbbing pain for 1 patient, and no pain for 1 other patient. At the one-month assessment, three patients described their pain as pulsing, two as tingling, one as pressure, and one as stabbing. Attempting to assess whether pain extended to other structures, it was possible to quantify that, at 48 h, only three people reported pain extending to other areas, and at one month postoperative, only one person reported pain extending to other regions. There were no statistically significant differences between groups (Figure 12).

**Figure 13:**
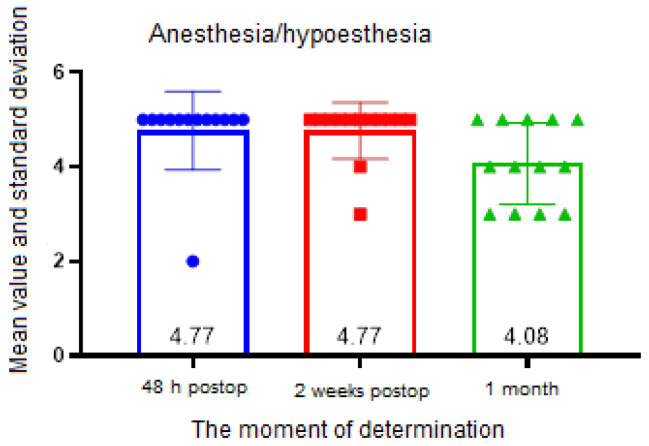
Anesthesia, hypoesthesia assessment.

In the Friedman test, there was a statistically significant difference between the average values of the ranks of the groups’ values in the presence of anesthesia/hypoesthesia (*p* = 0.006). However, Dunn’s test for group comparison revealed no statistically significant difference between any time points (Figure 13).

We tested the correlation between edema CD and pain intensity at 48 h, 2 weeks. and 1 month using the Spearman rank correlation test. There were no significant correlations between the variables at any time point. The correlation coefficient (r) was r = 0.41 at 48 h (*p* = 0.16), r = 0.34 at 2 weeks (*p* = 0.25), and r = 0.33 at 1 month (*p* = 0.25).

## 4. Discussion

The present study determined and assessed the most common symptoms associated with bimaxillary orthognathic surgery. We also found several correlations between the initial values and the values at different time intervals, the type of dentomaxillary anomaly and the tendency of changes of the monitored parameters. All three objective parameters assessed at the facial level showed significant differences in edema between the initial preoperative values and the postoperative values at 48 h. These differences were obvious and clinically confirmed during the patient’s examination by the noticeable increase in soft-tissue volume. Additionally, there were notable changes between the 48 h postoperative clinical assessment and the assessments at 2 weeks, 1 month, and 3 months, as clinically reflected by the remission of the inflammatory phenomena from one clinical control to the next. The edema was minimal at the 3-month postoperative follow-up, close to the initial values. Kwon et al. found that, at 6 and 21 months following surgery, no considerable alterations were seen, indicating that postponed soft-tissue modifications do not take place in patient populations who have undergone surgical procedures after surgically related facial swelling and edema had subsided, and the hard tissue beneath the soft tissue had healed [18].

Van der Vlis et al. assessed the volumetric analysis of postoperative edema after orthognathic surgery interventions, quantifying postoperative inflammatory changes and showed that postoperative edema decreased by 50% of the initial one after the 3rd week, and after 3 months, only 20% of the initial edema remained. The authors concluded that the rapid resolution of facial edema occurred in the first three postoperative weeks, but also its resolution continued between 6 and 12 months postoperatively [19], which we also encountered.

In the current study, the results were similar to those mentioned above. This study evaluated edema as the distance between the external canthus, gonion, tragus, cheilion, and gnathion of the eye. Postoperative edema decreased considerably from one clinical visit to the next, its value being maximal at the evaluation at 48 h postoperative and minimal at 3 months. These results are in concordance with the ones reported by Reategui et al., who revealed that the majority of facial edema disappeared within the initial month, with a considerable decline in edema occurring within 6 and 12 months later in patients following double jaw surgery [20].

Given the noticeable differences in postoperative edema severity among patients despite relatively similar treatments, it is indisputable that each patient’s unique reactivity has a considerable influence on this criterion. Furthermore, contrary to expectation, there was no relationship between the patient’s pain and the degree of edema. The insufficiency of the relationship between objective and subjective variables highlights the significance of each patient’s unique perception of pain, which varies while receiving the same treatment and is thereby not closely associated with any objective parameter assessed.

In terms of mouth opening amplitude, in our study, statistically significant differences were found between the values measured at the various established clinical controls. The skeletal class had no effect on the amplitude of mouth opening when the time variable was considered; however, the time of determination had a significant impact on the results. Clinically, there was a significant decrease in mouth opening amplitude at 2 weeks postoperative compared to the preoperative value, and a significant increase in mouth opening amplitude at subsequent clinical assessments. This parameter underlines the importance of physiotherapy after orthognathic surgery for temporomandibular joint rehabilitation and for overall patient comfort. Patients reported significant improvements in masticatory function beginning at two weeks after surgery and continuing for three months. All these patients underwent a period of presurgical orthodontic treatment aiming to decompensate the dental anomaly, which translates into poorer-than-before occlusion and masticatory function. The removal of the surgical splint was performed one month after surgery, which coincided with a high increase in patient comfort, oral hygiene possibilities, and masticatory function.

Bai et al. showed that short-term craniofacial function impairment in Skeletal Class III patients could be caused by orthognathic surgery, and individuals’ mouth openings were less than they had been preoperatively; however, over time, orthognathic surgery lead to more stable and symmetrical orofacial functions [21]. Meneses-Santos et al. showed the beneficial effects of low-level laser therapy in maximal mouth opening following orthognathic surgery [22]. Additionally, Joachim et al. observed a reduction in mouth opening amplitude. The majority of patients’ concerns following orthognathic surgery were nasal aspect and mouth openness [23]. The small amount of research with which our findings may be compared shows how little information there is in the literature evaluating opening amplitude. As a result, it might be regarded as an original feature of the current study.

Alyahya et al. reviewed the literature, and found that pre-emptive analgesics and low-level laser treatment significantly reduced pain within the first 48 h after orthognathic surgery, [24]. Opioid use was lower than what was anticipated following the orthognathic operation, however to prevent prescribing narcotics, prudence is required [25].

In this study, both the skeletal class and the time of determination influenced the width of the nose. Neither the time of determination nor the skeletal class had a significant influence on the values for the distance measured between the insertions of the nasal fins and the nostril diameter. Clinical examination, and a comparison of preoperative and postoperative photographs at 3 months revealed a widening of the interalar diameter.

Khamashta-Ledezma et al. reviewed the literature to assess nose modifications following maxillary orthognathic surgery, and demonstrated the broadening of the nares and the widening of the alar base after nearly all maxillary osteotomies. The nose width showed postoperative changes concerning the width of the nose, the exposure of the nostrils, the orientation of the columella, and the nasolabial angle [26]. van Loon et al. showed that the anterior translation of the maxilla and its clockwise inclination resulted in a significant increase in the volume of the upper lip and in the width of the alar portion [27].

The findings of this study confirm the existence of postoperative alterations at the level of the nose, particularly an increase in its width. These modifications are comparable to those observed in the previously described studies. A proper surgical strategy must be adopted if a nose widening is not necessary from a functional or aesthetical point of view. This might include strategically placed suture points after maxilla repositioning or even osteotomies at the level of the piriform aperture.

Regarding pain, in our study, the distribution of its intensity differed significantly between the moments of determination, particularly between the evaluations at 48 h and 1 month postoperative. There were no significant differences in pain radiation between the groups, and at the end of the study, at the 3-month postoperative evaluation, only 1 patient out of the 13 confirmed the presence of pain radiation. Except for one patient who described the pain as moderate, the patients at the 3-month postoperative follow-up showed minimal values on the intensity scale. Most patients described their pain as a constant pressure in the affected areas.

After the bimaxillary orthognathic procedure, Dadmehr et al. reported that the introduction of oral tizanidine was successful in lowering postoperative soreness [28]. Following orthognathic surgery, acute chronic postoperatively pain can be predicted using the pain catastrophizing scale and presurgical conditioned pain modulation [29]. In the current study, at the 48 h postoperative evaluation of the intensity rating scale, the highest value was 6, and at the 3-month follow-up, the presence of pain and intensity was minimal (below 3 on the intensity scale) except for one patient who scored the felt value as 5 on the intensity scale.

When analyzing the presence of anesthesia/hypoesthesia, there were significant differences between the average values of the ranks of the groups’ values, but no significant differences between the moments of determination. The statistical difference between the Friedman test, which found a statistically significant difference between the mean values of the ranks of the group values, and the Dunn test, which found no statistically significant difference at any time of the determination, is explained by the type of nonparametric tests used and an insufficient number of patients. Clinically, no patient reported a complete resolution of the neurosensorial phenomenon at the end of the study, with anesthesia/hypoesthesia present to varying degrees in each patient. Degala et al. showed that, following orthognathic surgery, the occurrence of a neurosensory impairment of the lower lip and chin was substantial, this being correlated with the operative expertise and intraoperative nerve contact, nevertheless, and the frequency of sensory return increased during the course of the follow-up period [30].

Kim et al. assessed the natural recovery of neurological damage after orthognathic surgery on the basis of subjective neurological assessment, and showed that sensory changes occurred in proportions of 55.7% at the chin level and 27.3% at the lip level. The altered neurosensorial sensation that may develop after orthognathic surgery is an unavoidable complication, but, with time, this may resolve spontaneously. In patients who also underwent a simultaneous genioplasty, the incidence of altered sensation was high, but not significantly associated with the patient’s age or with performing simultaneous maxillary surgery [31].

Yamamoto et al. evaluated tactile restoration following sagittal split ramus osteotomy, and explored the association between the degree of neurosensory disturbance and mandible migration length. They found that the occurrence of a neurosensory disruption in regards to tactile sensation may be higher in the category with more mandible advancement immediately postoperative [32].

Schlund et al. described a customized mandibular bilateral sagittal split osteotomy that protects the mandible lower margin, which causes a neurosensory disruption of the inferior alveolar nerve, which reduces the likelihood of postoperative hypoesthesia [33].

Hanfesh et al. showed that three months was a sufficient healing duration to fully re-establish neurological feeling after bilateral sagittal split osteotomy [34].

Thiem et al. observed that long-term difficulties after orthognathic surgery arose when they assessed intraoperative and early postoperative consequences, delayed outcomes, and patients’ average contentment, with hypoesthesia of the lower lip being an encountered side effect [35]. However, Ahmad et al. described that following mandible orthognathic surgery, individuals who experienced lower lip neuropathy showed no negative effects on their comfort or quality of life [36].

As far as we are aware, no other investigations have simultaneously followed all the research parameters that were assessed in this research. Even if a similar study has been performed, the findings from our study are still highly significant and important because every surgical team has its own treatment regimen and surgical approach, and diverse populations respond differently to the same surgical procedure.

The current study was carried out in a clinic that treats a sizable number of orthognathic patients annually. Consequently, the team has a richness of experience. The parameters that were assessed throughout this pilot study and their values may be used to compare the recovery of patients treated using this protocol with those treated using other protocols, allowing for the authors and readers from other centers to modify their surgical approaches for better outcomes, particularly in terms of patient comfort.

### 4.1. Limitations and Strengths

The small sample, brief follow-up period, and reliance on the examiner’s objectivity for the accurate measurements of the monitored parameters were the study’s limitations.

### 4.2. Implications for Future Research

Given the extensive tissue manipulation involved in these surgical procedures, our research indicates that the recovery time following surgical interventions to treat dental–maxillary anomalies should receive special attention. This is because the recovery process is complicated and prolonged and incorporates a number of the patients’ objective and subjective signs. The healing process must be monitored and evaluated to promote proper and harmonious healing, the remission of inflammatory, nervous, and painful phenomena, and the avoidance of further complications. The recovery time directly affects the patient’s quality of life. To increase the power of this clinical trial, it would be necessary to enroll a larger number of patients in the study, and the monitored parameters need to be followed for a longer period.

## 5. Conclusions

The complexity and extent of orthognathic surgery requires an increased awareness of patients regarding everything that this type of surgery entails, from the aesthetic, functional, and social implications to the extent of the treatment and postoperative care. The recovery period after orthognathic surgery can last up to six months. During the recovery period, the most frequently associated objective and subjective signs are edema, hematoma, mouth opening limitations, pain, anesthesia/hypoesthesia, with edema being maximal at 48–72 h postoperatively. At the three-month postoperative follow-up, the patients showed minimal values of pain on the intensity scale, with the most common type of pain being the feeling of continuous pressure. The resolution of the neurosensorial phenomenon was incomplete at three months after surgery, and present in all patients in different degrees. After bimaxillary orthognathic surgery, soft-tissue changes occurred with a direct influence on facial aesthetics. These findings provide valuable insight into the field, helping practitioners in better developing their intra- and postoperative strategies to obtain the best results and minimize patients’ discomfort.

## Figures and Tables

**Table 1:** Patient characteristics.

Number	Age	Sex	Skeletal Class
1	18	Female	III
2	29	Female	III
3	29	Female	II
4	27	Female	II
5	24	Male	II
6	22	Male	III
7	28	Female	II
8	22	Male	III
9	30	Female	II
10	25	Female	II
11	29	Male	III
12	21	Female	III
13	35	Male	III

## Data Availability

The data presented in this study are available from the corresponding author upon reasonable request.

## References

[1] Roslan A.A., Rahman N.A., Alam M.K. (2018). Dental Anomalies and Their Treatment Modalities/Planning in Orthodontic Patients. J. Orthod. Sci..

[2] Wiechens B., Quast A., Klenke D., Brockmeyer P., Schliephake H., Meyer-Marcotty P. (2022). Evaluation of Occlusal Function during Orthognathic Therapy: A Prospective Clinical Trial Using a Digital Registration Method. J. Orofac. Orthop. Fortschr. Kieferorthopädie.

[3] Arnett G.W., D’Agostino A., Grendene E., McLaughlin R.P., Trevisiol L. (2022). Combined Orthodontic and Surgical Open Bite Correction: Principles for Success. Part 2. Angle Orthod..

[4] Daluz A.D.J., da Silva T.V.S., Tôrres B.O., Costa D.F.N., Santos L.A.D.M. (2022). Long-Term Airway Evolution after Orthognathic Surgery: Systematic Review. J. Stomatol. Oral Maxillofac. Surg..

[5] Applied Facial Anatomy—ClinicalKey. https://www.clinicalkey.com/#!/content/book/3-s2.0-B9780323358767000029?scrollTo=%23hl0000165.

[6] Moorhead A., Serra M. (2022). Le Fort Osteotomy.

[7] Monson L. (2013). Bilateral Sagittal Split Osteotomy. Semin. Plast. Surg..

[8] Lu Y., Zhang W., Zhao B., Liu Y. (2022). Vertical Control of a Severe Hyperdivergent Skeletal Class II Malocclusion with Steep Posterior Occlusal Plane in a Camouflage Case. Medicina.

[9] Hamdy Mahmoud M., Ismail Elfaramawi T. (2022). Maxillary Stability in Patients with Skeletal Class III Malocclusion Treated by Bimaxillary Orthognathic Surgery: Comparison of Mandible-First and Maxilla-First Approaches in a Randomised Controlled Study. Br. J. Oral Maxillofac. Surg..

[10] Woods M.G. (2022). An Assessment of Surgical and 10-Year Follow-up Vertical Changes after Contemporary Class II and III Orthognathic Surgery. Am. J. Orthod. Dentofac. Orthop..

[11] Altman J.I., Oeltjen J.C. (2007). Nasal Deformities Associated with Orthognathic Surgery: Analysis, Prevention, and Correction. J. Craniofac. Surg..

[12] Barbosa L.M., de Luna Gomes J.M., Laureano Filho J.R., do Egito Vasconcelos B.C., Dantas Moraes S.L., Pellizzer E.P. (2022). Does the Use of Low-Level Light Therapy Postoperatively Reduce Pain, Oedema, and Neurosensory Disorders Following Orthognathic Surgery? A Systematic Review. Int. J. Oral Maxillofac. Surg..

[13] De Lima V.N., Lemos C.A.A., Faverani L.P., Santiago Júnior J.F., Pellizzer E.P. (2017). Effectiveness of Corticoid Administration in Orthognathic Surgery for Edema and Neurosensorial Disturbance: A Systematic Literature Review. J. Oral Maxillofac. Surg..

[14] Glass G.E., Waterhouse N., Shakib K. (2016). Hilotherapy for the Management of Perioperative Pain and Swelling in Facial Surgery: A Systematic Review and Meta-Analysis. Br. J. Oral Maxillofac. Surg..

[15] Domínguez Camacho A., Velásquez S.A., Benjumea Marulanda N.J., Moreno M. (2020). Photobiomodulation as Oedema Adjuvant in Post-Orthognathic Surgery Patients: A Randomized Clinical Trial. Int. Orthod..

[16] Sirintawat N., Sawang K., Chaiyasamut T., Wongsirichat N. (2017). Pain Measurement in Oral and Maxillofacial Surgery. J. Dent. Anesth. Pain Med..

[17] Agbaje J., Luyten J., Politis C. (2018). Pain Complaints in Patients Undergoing Orthognathic Surgery. Pain Res. Manag..

[18] Kwon J.-J., Kang Y.-H., Hwang D.-S. (2022). Delayed Soft Tissue Changes after Clockwise Rotation of the Maxillo-Mandibular Complex. J. Craniofac. Surg..

[19] van der Vlis M., Dentino K.M., Vervloet B., Padwa B.L. (2014). Postoperative Swelling After Orthognathic Surgery: A Prospective Volumetric Analysis. J. Oral Maxillofac. Surg..

[20] Reategui A., Phillips S., Dinis J., Junn A., Parsaei Y., Yang J., Lopez J., Steinbacher D.M. (2022). Postoperative Edema Resolution Post-Orthognathic Triple Jaw Surgery: A Three-Dimensional Volumetric Analysis. J. Craniofac. Surg..

[21] Bai Y., Tang Y., Ren M., Wang M., Zhao W., Zeng T., Liu F., Zhu S. (2022). Orofacial Myofunctional Changes in Skeletal Class III Patients after Bimaxillary Orthognathic Surgery. J. Plast. Reconstr. Aesthet. Surg..

[22] Meneses-Santos D., Costa M.D.M.A., Inocêncio G.S.G., Almeida A.C., Vieira W.A., Lima I.F.P., Paranhos L.R. (2022). Effects of Low-Level Laser Therapy on Reducing Pain, Edema, and Trismus after Orthognathic Surgery: A Systematic Review. Lasers Med. Sci..

[23] Joachim M.V., Richter D.E., Mohana A., Labeeb M., Abdelraziq M., Abu El-Naaj I. (2021). Quality of Life After Class III Repair Orthognathic Surgery: Five-Year Retrospective Study. J. Craniofac. Surg..

[24] Alyahya A., Aldubayan A., Swennen G.R.J., Al-Moraissi E. (2022). Effectiveness of Different Protocols to Reduce Postoperative Pain Following Orthognathic Surgery: A Systematic Review and Meta-Analysis. Br. J. Oral Maxillofac. Surg..

[25] Bousquet B., Green M.A., Caillouette C.N., Simon J., Padwa B.L., Resnick C.M. (2022). How Much Opioid Medication Do Patients Need After Orthognathic Surgery?. J. Oral Maxillofac. Surg..

[26] Khamashta-Ledezma L., Naini F.B., Manisalı M. (2017). Review of Nasal Changes with Maxillary Orthognathic Surgery. J. Istanb. Univ. Fac. Dent..

[27] Van Loon B., van Heerbeek N., Bierenbroodspot F., Verhamme L., Xi T., de Koning M.J.J., Ingels K.J.A.O., Bergé S.J., Maal T.J.J. (2015). Three-Dimensional Changes in Nose and Upper Lip Volume after Orthognathic Surgery. Int. J. Oral Maxillofac. Surg..

[28] Dadmehr S., Shooshtari Z., Alipour M., Eshghpour M., Shaban B., Vaezi T., Samieirad S. (2022). Is Preemptive Oral Tizanidine Effective on Postoperative Pain Intensity after Bimaxillary Orthognathic Surgery? A Triple-Blind Randomized Clinical Trial. World J. Plast. Surg..

[29] Takashima K., Oono Y., Takagi S., Wang K., Arendt-Nielsen L., Kohase H. (2022). Acute Postoperative Pain after Orthognathic Surgery Can Be Predicted by the Preoperative Evaluation of Conditioned Pain Modulation and Pain Catastrophizing. Pain Rep..

[30] Degala S., Shetty S.K., Bhanumathi M. (2015). Evaluation of Neurosensory Disturbance Following Orthognathic Surgery: A Prospective Study. J. Maxillofac. Oral Surg..

[31] Kim Y.-K., Kim S.-G., Kim J.-H. (2011). Altered Sensation After Orthognathic Surgery. J. Oral Maxillofac. Surg..

[32] Yamamoto T., Fujii-Abe K., Fukayama H., Kawahara H. (2017). Hypoesthesia Associated with Mandibular Movement after Sagittal Split Ramus Osteotomy. Oral Maxillofac. Surg..

[33] Schlund M., Grall P., Ferri J., Nicot R. (2022). Effect of Modified Bilateral Sagittal Split Osteotomy on Inferior Alveolar Nerve Neurosensory Disturbance. Br. J. Oral Maxillofac. Surg..

[34] Hanfesh A., Salma R.G., Al Mutairi K., AlShiha S.K., Al Otaibi S. (2021). The Neurosensory Deficit of Inferior Alveolar Nerve Following Bilateral Sagittal Split Osteotomy: A Prospective Study. Oral Maxillofac. Surg..

[35] Thiem D.G.E., Schneider D., Hammel M., Saka B., Frerich B., Al-Nawas B., Kämmerer P.W. (2021). Complications or Rather Side Effects? Quantification of Patient Satisfaction and Complications after Orthognathic Surgery—A Retrospective, Cross-Sectional Long-Term Analysis. Clin. Oral Investig..

[36] Ahmad Z., Breeze J., Williams R. (2018). Numbness of the Lower Lip Does Not Adversely Affect Quality of Life or Patients’ Satisfaction after Mandibular Orthognathic Surgery. Br. J. Oral Maxillofac. Surg..

